# Supplementary material for: Factors associated with the lethality of patients hospitalized with severe acute respiratory syndrome due to COVID-19 in Brazil
Source: PLOS Glob Public Health. 2022 Apr 13;2(4):e0000200. doi: 10.1371/journal.pgph.0000200 (PMC10021557; doi:10.1371/journal.pgph.0000200)
Supplement: Attachment — Submitted filename: RESPONSE TO REVIEWERS 02.03.2022.docx [file pgph.0000200.s001.docx]

**RESPONSE TO REVIEWERS**

To the **PLOS Global Public Health** editorial team, regarding the PGPH-D-21-00716 manuscript - Factors associated with the lethality of patients hospitalized with severe acute respiratory syndrome due to COVID-19 in Brazil.

We would like to thank the reviewers for their precious contributions. We would like to inform you that all the requested changes have been carried out, as detailed below:

1. Are the data from the URL <https://opendatasus.saude.gov.br/> third party data? (i.e. not owned or collected by the author). If so, please confirm that the authors had no special access or privileges to the data that other researchers would not have.

*-* We reinforce that this is data from third parties, that is, not owned or collected by the author. We emphasize that the authors had no special access or privileges to the data that other researchers would not have. These are official data from the official website of the Brazilian government.

2. Google drive is not considered an acceptable repository for lasting data storage. We kindly ask the authors to deposit the data into a stable, publicly accessible repository and update their Data Availability Statement to provide the final DOI. For a list of our recommended repositories, please view our website here: <https://journals.plos.org/plosone/s/recommended-repositories>.

- The data are freely available for public access in the GOV.BR repository. It is maintained by the Brazilian government, with access available to anyone, without any cost and restrictions. The data are publicly available from <https://opendatasus.saude.gov.br/dataset/srag-2020>
